# 7,8-dihydroxyflavone enhances long-term spatial memory and alters brain volume in wildtype mice

**DOI:** 10.3389/fnsys.2023.1134594

**Published:** 2023-03-15

**Authors:** Florence Rawlings-Mortimer, Alberto Lazari, Cristiana Tisca, Mohamed Tachrount, Aurea B. Martins-Bach, Karla L. Miller, Jason P. Lerch, Heidi Johansen-Berg

**Affiliations:** Nuffield Department of Clinical Neurosciences, Wellcome Centre for Integrative Neuroimaging, Oxford, United Kingdom

**Keywords:** 7,8-dihydroxyflavone, flavonoids, Morris water maze, spatial memory, structural MRI, T2-weighted imaging

## Abstract

**Introduction:** 7,8-dihydroxyflavone (7,8-DHF) is a low molecular weight compound that can cross the blood brain barrier and has been implicated in numerous functions and behaviours. It is thought to have neuroprotective capability and has been shown to alleviate symptoms in a wide range of diseases.

**Methods:** 7,8-DHF was administered systemically to wildtype mice during Morris water maze training. Long-term spatial memory was assessed 28 days later. *Ex-vivo* T2-weighted (T2w) imaging was undertaken on a subset of these mice to assess brain-wide changes in volume.

**Results:** We found that systemic 7,8-DHF administration during the training period enhanced spatial memory 28 days later. Volumetric changes were observed in numerous brain regions associated with a broad range of functions including cognition, sensory, and motor processing.

**Discussion:** Our findings give the first whole brain overview of long-term anatomical changes following 7,8-DHF administration providing valuable information for assessing and understanding the widespread effects this drug has been shown to have in behaviour and disease.

## Introduction

Several low molecular weight compounds have been developed and proposed to be tyrosine kinase B (TrkB) receptor agonists that can mimic brain derived neurotrophic factor (BDNF; Jang et al., 2010; Massa et al., 2010). Although much research has linked these drugs to BDNF agonism, there is evidence that suggests 7,8-dihydroxyflavone (7,8-DHF) may work through alternative mechanisms (Pankiewicz et al., 2021). 7,8-DHF is a rare flavonoid lacking B-ring oxygenation (Liu et al., 2016). Flavonoids such as anthocyanins naturally occur in many fruits and vegetables including blueberries and strawberries. Correlations have been shown between the number of anthocyanin compounds in the cortex and hippocampus and performance on the Morris water maze (MWM) test of spatial memory in rats supplemented with blueberries (Andres-Lacueva et al., 2005). Likewise, dietary strawberry supplementation was found to improve virtual MWM and word recognition performance in older adults (Miller et al., 2021).

7,8-DHF administration has been shown to alleviate symptoms in a wide range of brain disorders. Decreased Alzheimer’s disease (AD) related pathology including reduced Aβ plaque deposition and protection against reduced dendritic arbour complexity were seen with 7,8-DHF administration in the 5XFAD mouse model (Aytan et al., 2018). In this same model, 7,8-DHF administration has been shown to rescue memory impairment in the spontaneous alternation Y-maze and increase levels of BACE1 (Devi and Ohno, 2012), as well as improved short-term MWM memory (Zhang et al., 2014). 7,8-DHF has been shown to have beneficial effects in other AD animal models. It improved short-term MWM memory in CaM/Tet-DT_A_ mouse model (Castello et al., 2014) and improved MWM learning and short-term memory when given chronically prior to training in the Tg2576 AD mouse model (Gao et al., 2016). It “mimicked” the positive effect of social interaction in the APP/PS1 AD mouse model (Hsiao et al., 2014), and improved object recognition in the APPswe/PS1dE9 model of AD (Bollen et al., 2013). 7,8-DHF has also been found to improve symptoms in animal models of Parkinson’s disease (Nie et al., 2019), amyotrophic lateral sclerosis (ALS; Korkmaz et al., 2014), Huntington’s disease (Jiang et al., 2013), traumatic brain injury (Agrawal et al., 2015), post-traumatic stress disorder (PTSD; Andero et al., 2012; Wang et al., 2022), depression (Amin et al., 2020), Wolfran syndrome (Seppa et al., 2021), Down syndrome (Stagni et al., 2017), and an *in vitro* model of ischemic stroke (Zhou et al., 2022).

While there is growing evidence on the positive behavioural effects of 7,8-DHF, and emerging insight on cellular correlates, research investigating its impact on brain volume is limited. Given interest in the potential therapeutic benefits of 7,8-DHF, understanding its brain-wide effects, which could mediate behavioural improvements, is critical. Magnetic resonance imaging (MRI) is a useful technique in this respect, as it can give a brain-wide analysis of changes in structure and volume and can be translated to clinical settings. For example, one previous study demonstrated increase in functional MRI measures of hippocampal functional connectivity associated with effects of 7,8-DHF on exercise-mediated recovery in a mouse model of traumatic brain injury (Krishna et al., 2017). However, there has not yet been investigation of the effect of 7,8-DHF on brain-wide changes in volume.

In the current study we assessed the effect of 7,8-DHF administration on long-term spatial memory in mice and evaluated effects on brain volume using post-mortem whole-brain anatomical T2-weighted (T2w) imaging. Superior resolution and contrast can be gained by using ex-vivo MRI (Lerch et al., 2012).

## Materials and methods

### Experimental animals

All experiments were approved under the Animals (Scientific Procedures) Act 1986. 25 male and 29 female C57BL6J wildtype mice aged 14–16 weeks were housed in groups of 2–5 under a 12-h light/dark cycle and provided with ad libitum access to food and water. Behavioural training and testing were performed during the light phase at the same time each day. Male experimenters have been shown to produce increased stress responses in rodents (Sorge et al., 2014; Faraji et al., 2022). Therefore, all experiments were undertaken by the same female experimenter. Animal handling techniques that reduce stress and promote animal welfare were also used (Sensini et al., 2020).

### Morris water maze

The Morris water maze (MWM) is the most widely used behavioural test for assessing spatial memory (Morris, 1981). This is advantageous as behavioural performance can be easily compared between studies. There are many other benefits of this test including the uniformly motivating aspect of swimming in water with no need for dietary food or water restriction (Vorhees and Williams, 2014). The MWM also requires minimal training with low subject dropout compared to other learning paradigms (Vorhees and Williams, 2014). The main disadvantage is that it can potentially be stressful for the mice (Vorhees and Williams, 2014), although arguably less stress is caused compared to alternative tests that use prolonged dietary restriction. Steps taken to limit stress in this study included using low level lighting and limiting the number of trials to three on the first day of exposure to the water. Stress caused by the experimenter was also limited as outlined in the section above.

The MWM, diameter 2 m, was filled with water to a depth of ~0.29 m. To escape from the water, the mice were required to find a hidden platform (diameter 21 cm) with fixed location and submerged approximately 1 cm below the water surface. The swim paths of the mice were recorded and tracked using Watermaze software (Actimetrics, Wilmette, USA). During the training period the mice received four trials per day for a total of 7 days. In each trial they were placed into the pool at one of eight different starting points in a randomly selected order. The mice had a maximum of 90 s to find the platform and once found remained on the platform for 15 s. If unsuccessful, the mice were guided gently to the platform’s location. Twenty-eight days later the mice underwent a probe test. During the probe test the platform was removed and the mice swam freely for 45 s. The percentage of time they spent in the four quadrants of the maze, the time to reach the platform, and the number of platform crossings, along with their average speed were recorded.

### Drug preparation and administration

7,8-dihydroxyflavone hydrate (7,8-DHF; Merk Life Sciences, Gillingham, UK) was dissolved in 17% DMSO/PBS. Mice received one intraperitoneal (i.p) injection of 7,8-DHF (5 mg/kg) or vehicle (controls) immediately following each MWM training session (seven in total). We chose this dose of 7,8-DHF as it has been widely used and shown to improve symptoms in a number of disease models (Zeng et al., 2012; Zhang et al., 2014; Krishna et al., 2017; Stagni et al., 2017).

### MRI data: sample preparation

Following the MWM probe test, 24 randomly selected mice (DHF *n* = 12; *f* = 9/*m* = 3, vehicle *n* = 12; *f* = 7/*m* = 5) were anaesthetized using pentobarbital and perfused with 30 ml of PBS containing Gadovist (2 mM, Bayer, Berlin, Germany), followed by 30 ml of 4% paraformaldehyde containing Gadovist (2 mM, Bayer) and Heparin (1 μl/ml, Wockhardt, Wrexham, UK) at a flow rate of 1 ml/min (Cahill et al., 2012). The skulls containing the brains were removed and postfixed in 4% PFA with 2 mM Gadovist for 36 h at 4°C. They were then transferred into PBS with 0.02% sodium azide and Gadovist and stored at 4°C. Prior to scanning, the skulls were transferred into a 15 ml falcon tube containing fluorinert (3M) and placed in a vacuum pump to remove any potential bubbles.

### MRI data: acquisition

The MRI data was collected on a BioSpec 70/20 (7T field strength, 20 cm bore diameter) small animal MR system with a Paravision 360 console (Bruker BioSpin MRI, Ettlingen, Germany). T2w anatomical MRI was acquired using TurboRARE 3D with the following parameters: TR = 350 ms, echo spacing = 12 ms, six echoes, TEeff = 36 ms, field of view (FOV) 24 × 9.6 × 12 mm, matrix size 400 × 160 × 200, resolution = 60 × 60 × 60 μm, scan time was 33 min.

### MRI data: processing

The Pydpiper toolkit was used to register and segment the T2w images (Friedel et al., 2014). The images were registered linearly and nonlinearly, resampled, and averaged to create unbiased population sample study templates (Chakravarty et al., 2013). The volumetric information was extracted from the high-resolution T2w images by calculating the Jacobian determinant of the deformations (Chung et al., 2001). Segmentation was undertaken using an anatomical atlas consisting of 182 structures across the whole brain (Dorr et al., 2008; Richards et al., 2011; Ullmann et al., 2013; Steadman et al., 2014; Qiu et al., 2018).

### MRI and behavioural data: statistical analysis

MWM behavioural data were assessed for normal distribution using the Shapiro-Wilk test and for homogeneity of variances using Levene’s test. If the data was found to have normal distribution and homogeneity of variances then two-way mixed ANOVA, independent student’s *t*-test or Pearson’s r test were used. If the assumptions for parametric tests were not met then a Wilcoxon rank sum test was used. Data were analysed with R studio (version 2021.09.2). Data are presented as mean ± standard deviation (SD) or standard error of the mean (SEM); graphs were generated in GraphPad Prism (version 9.3.0).

Statistical analyses of T2w data were run using the RMINC and MRIcrotome toolkits^1^. False discovery rate (FDR) was used to control for multiple comparisons on all MRI analysis undertaken (Genovese et al., 2002). Whole brain voxelwise and region of Interest (ROI) analyses were run on the volumetric data. Voxewise analyses used whole-brain Jacobian determinant images, while ROI analysis calculated and used the volume of all brain areas, which were segmented using an anatomical atlas consisting of both white and grey matter regions of interest across the whole brain (Dorr et al., 2008; Richards et al., 2011; Ullmann et al., 2013; Steadman et al., 2014). A linear mixed effect model was then fit either at each voxel or at each ROI, testing for effects of drug group. Two mice were excluded from the T2w analysis due to incomplete Jacobian outputs. Voxelwise clusters showing significant drug-related effects were then used to extract average Jacobian values across voxels within the cluster for each mouse. These Jacobian values were then correlated with the respective behavioural performance of each mouse (as measured by the percentage of time spent in the target quadrant) through a Pearson’s r two-tailed test.

## Results

### 7,8-DHF administration enhances long-term spatial memory

**Figure 1:**
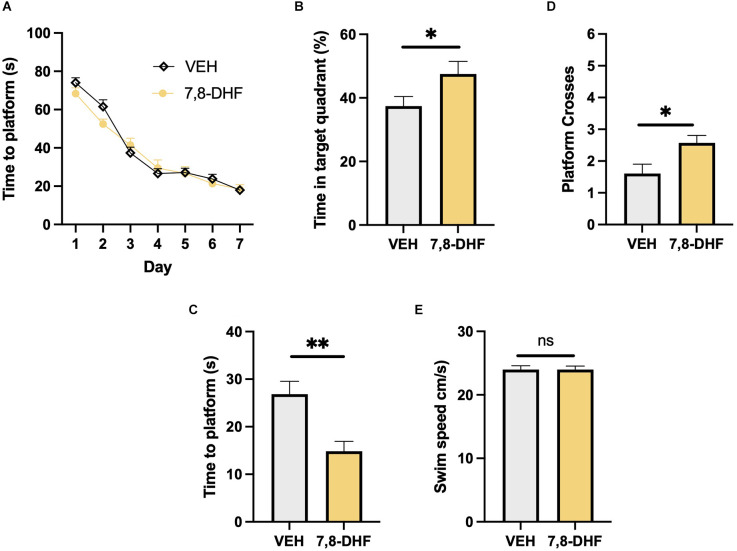
Long-term memory performance in the Morris water maze. **(A)** Both the mice that received 7,8-DHF and vehicle following each training session successfully learnt the location of the platform during the training period. **(B)** The 7,8-DHF group spent a greater percentage of time in the target quadrant during the 28-day probe test. **(C,D)** The 7,8-DHF group found the platform quicker and crossed it more often than the controls. **(E)** Swim speed was equivalent between groups . Data presented mean ± SEM. *< 0.05, **< 0.01, ns: not significant.

Both the mice that received 7,8-DHF (*n* = 21 *f* = 13/*m* = 8) and the controls (*n* = 33 *f* = 16/*m* = 17) that received vehicle successfully learnt the location of the platform during the 7 days of training. Time taken to find the platform was decreased in both groups indicating successful spatial memory acquisition (Two-way mixed ANOVA; *F*_2,52_ = 123.82, *P* < 0.001, Figure 1A) with no difference between groups (*F*_2,52_ = 0.44, *p* > 0.05) or group × day interaction (*F*_2,52_ = 1.68, *p* > 0.05). During the probe test 28 days later, the mice that had received 7,8-DHF during training spent a greater percentage of time in the target quadrant where the platform had previously been located compared to mice that received vehicle (Student’s *t* test; *t* = 2.04, df = 52, *p* < 0.05, Figure 1B) indicating enhanced long-term spatial memory. This measure is a percentage comparing the mouse’s preference for each of the four quadrants and is therefore independent of swim speed. The 7,8-DHF group also found the location where the platform had previously been located quicker (Wilcoxon rank sum test; *W* = 192, *z* = −2.73, *p* < 0.01, Figure 1C) and made a greater number of crossings of the platform area compared with the controls (Wilcoxon rank sum test; *W* = 498, *z* = −2.73, *p* < 0.01, Figure 1D). There was no difference in the swim speed between groups during the probe test (Student’s *t* test; *t* = 0.001, df = 52, *p* > 0.05, Figure 1E) indicating that locomotion was not altered and therefore had no impact on the behavioural measures of spatial memory assessed in this experiment.

### 7,8-DHF administration causes widespread neuroanatomical volume changes

A voxelwise analysis tested for whole-brain volumetric bidirectional changes using Jacobian determinant images in mice that had received 7,8-DHF (*n* = 10; *f* = 8/*m* = 2) during the MWM training period compared with those that received vehicle (*n* = 12; *f* = 7/*m* = 5). Widespread volumetric changes were observed (*t*_peak_ = 4.21, DF = 1,20, *q* < 0.05, FDR corrected). However, the average Jacobian values across all significant voxelwise clusters for each subject in the 7,8-DHF group were not found to correlate with the percentage of time spent in the target quadrant of the MWM during the 1-month probe test (Pearson’s *r* = −0.1 *p* > 0.05).

**Figure 2:**
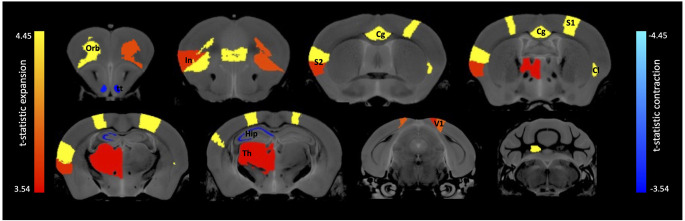
Brain regions showing volume expansion and contraction following 7,8-DHF administration. Anatomical labels: Cg, cingulate cortex; Cl, claustrum; Hip, hippocampus; In, insular cortex; Orb, orbital cortex; S1, primary somatosensory; S2, secondary somatosensory; Th, thalamus; tt, tenia tecta; V1, primary visual.

A region of interest (ROI) analysis tested for volumetric changes in the 7,8-DHF (*n* = 10; *f* = 8/*m* = 2) group compared with the controls (*n* = 12; *f* = 7/*m* = 5) in 99 ROIs covering cortical and subcortical brain regions. Results revealed significant changes in volume across a number of GM regions (*t*_peak_ = 4.45, DF = 1,20, *q* < 0.01, FDR corrected, Figure 2), with some regions larger in volume (Table 1) and some regions smaller in volume (Table 2) in the 7,8-DHF group compared to controls. Larger areas included the cingulate cortex area 24a (*q* = 0.001), the left fastigial nucleus (*q* = 0.003), the right ventral claustrum (*q* = 0.007), left insular region (*p* = 0.046), right lateral orbital cortex (*q* = 0.007), right posterior parietal cortex (*q* = 0.002), the right forelimb (*q* = 0.002), and hindlimb primary somatosensory cortex (*q* = 0.001). See Table 1 for a full list of structures. Other ROIs showed a smaller volume in the 7,8-DHF group, including the right (*q* = 0.05) and left (*q* = 0.05) ventral tenia tecta and the left stratum lucidum hippocampus (SLu *q* = 0.05) and the cornu ammonis 1 (CA1) pyramidal layer (*q* = 0.05).

**Table 1:** Brain structures showing an increase in volume following 7,8-DHF administration.

Structure	*t*-value	*q*-value
right Cingulate cortex: area 24a	6.2642	0.0007
right Primary somatosensory cortex: hindlimb region	6.0495	0.0007
left Cingulate cortex: area 24a	5.9044	0.0007
left Primary somatosensory cortex: hindlimb region	5.8869	0.0007
right Primary somatosensory cortex: forelimb region	5.4083	0.0017
right Parietal cortex: posterior area: rostral part	5.2424	0.0021
left fastigial nucleus	5.0128	0.0031
left Secondary somatosensory cortex	4.6290	0.0067
right Claustrum: dorsal part	4.5240	0.0073
right Claustrum: ventral part	4.4806	0.0073
left Lateral orbital cortex	4.4517	0.0073
right Primary visual cortex: monocular area	3.7638	0.0333
left Secondary visual cortex: mediolateral area	3.7348	0.0333
right Lateral orbital cortex	3.6636	0.0365
left Insular region: not subdivided	3.5306	0.0464
left thalamus	3.3855	0.0494
right Secondary visual cortex: mediolateral area	3.4469	0.0494
left Primary somatosensory cortex: upper lip region	3.3382	0.0494
right striatum	3.2612	0.0540
left Claustrum: ventral part	3.2782	0.0540
right Cingulate cortex: area 32	3.1355	0.0675
left Primary somatosensory cortex: forelimb region	3.0806	0.0675
right nucleus interpositus	3.0349	0.0679
left Ectorhinal cortex	3.0429	0.0679
left Lateral parietal association cortex	3.0342	0.0679
left striatum	2.9832	0.0739
right fastigial nucleus	2.9342	0.0800

**Table 2:** Brain structures showing a decrease in volume following 7,8-DHF administration.

Structure	*t*-value	*q*-value
right Ventral tenia tecta	−3.3565	0.0494
left Ventral tenia tecta	−3.4216	0.0494
left Stratum lucidum hippocampus	−3.3599	0.0494
left CA1 pyramidal	−3.3812	0.0494
right Secondary auditory cortex: dorsal area	−3.1105	0.0675
left inferior olivary complex	−3.0842	0.0675
left CA2 oriens layer	−3.1067	0.0675

## Discussion

We found that administering 7,8-DHF during MWM training enhanced long-term spatial memory retention 28 days later and increased brain volume across widespread brain regions. This provides the first evidence of brain-wide effects of 7,8-DHF and adds to a growing literature suggesting its positive effects on brain and behaviour. To the best of our knowledge, this is the first study reporting long-term benefits of 7,8-DHF on spatial memory in the MWM. We did not test the mice at shorter time intervals of 24 h or less. However, previous research has reported improved short-term MWM performance following 7,8-DHF administration in various disease models (Andero et al., 2012; Castello et al., 2014; Zhang et al., 2014; Gao et al., 2016; Tan et al., 2016; Akhtar et al., 2021; Seppa et al., 2021) and in aged rats (Zeng et al., 2012).

We found that administering 7,8-DHF resulted in changes to brain volume in a wide range of brain areas including area 24 of the cingulate cortex, lateral orbital cortex, the claustrum, insular region, parts of the somatosensory cortex, the striatum, and deep cerebellar nuclei including the fastigial nucleus. 7,8-DHF was also found to decrease volume in other regions including the ventral tenia tecta and regions of the hippocampus. We did not, however, find any significant correlation with the percentage of time spent in the target quadrant, time to reach the target platform or number of platform crosses during the MWM probe test. Given the broad range of diseases and behaviours that 7,8-DHF has been associated with, it is perhaps not surprising that numerous brain areas were found to be altered by its administration. Further research will be needed to determine the behaviours these brain volumes changes are associated with. We have outlined below previous literature indicating potential roles of these brain regions in behaviour and disease to inspire future work using 7,8-DHF.

The cingulate cortex has been implicated in a wide range of brain functions including the recall of long-term spatial and object recognition memory (Pezze et al., 2017; Rolls, 2019a). Afferents of the cingulate cortex area 24a include the orbital medial prefrontal cortex (mPFC), the claustrum, CA1 and ventral subiculum of the hippocampus, the basolateral amygdala (BLA), the thalamus, hypothalamus and brainstem (Fillinger et al., 2017) whereas efferents include the claustrum, striatum, BLA, lateral septum, orbital cortex, thalamus, hypothalamus, brainstem, and light inputs to the dorsal tenia tecta (Fillinger et al., 2018). The cingulate cortex has been proposed to predict AD progression (Lee et al., 2020). Neuropsychiatric symptoms of depression and AD have also been associated with the cingulate and orbitalfrontal cortex (Rolls, 2019b; Rolls et al., 2019; Chen et al., 2021). The orbitalfrontal cortex is also thought to play a role in motivated decision making (Zimmermann et al., 2017). 7,8-DHF was found to rescue impaired reinforcement motivated decision making in mice with a knock-down of BDNF in the orbitofrontal cortex (Zimmermann et al., 2017).

It has been suggested that the claustrum is an important structure for synchronizing different modalities including cognitive, motor, and perceptual (Crick and Koch, 2005). It has also been proposed that the claustrum is important in salience-guided orienting. It may work to integrate information from its connections with the mPFC, medial-dorsal thalamus, and BLA to focus attention and coordinate responses of its output structures such as the motor and sensory cortex (Smith et al., 2019). The claustrum has been linked to a number of disorders including AD, depression, and Parkinson’s disease (PD; Nikolenko et al., 2021). The insular cortex is another well connected brain region that has been shown to have numerous functions including processing of visceral sensations, somatosensory, pain, auditory, taste, experiencing of emotions such as disgust, sadness and happiness, empathy as well as cognitive processes such as decision making and attention (Uddin et al., 2017; Evrard, 2019). It is also thought to have a role in recognition memory and regulating cardiac function (Bermudez-Rattoni, 2014; Oppenheimer and Cechetto, 2016). The insular cortex has been implicated in depression, AD, and PD (Sprengelmeyer et al., 2011; Fathy et al., 2020).

The volume of striatum and fastigial nucleus in the cerebellum was found to be increased following 7,8-DHF administration. These brain regions have traditionally been associated with motor function and previous research indicates involvement of 7,8-DHF in the improvement of motor outcomes. For example, 7,8-DHF was found to protect against loss of dopaminergic neurons in the substantia nigra and striatum and improve open field locomotion in a rat model of Parkinson’s disease (Nie et al., 2019). Improvement in motor deficits was seen in an ALS mouse model along with increased numbers of motor neurons and dendritic spines in the lumbar spinal cord (Korkmaz et al., 2014). 7,8-DHF was found to enhance the survival of cultured motor neurons (Tsai et al., 2013) and improved motor function in a mouse model of Huntington’s disease (Jiang et al., 2013). Increased mitochondrial content was also observed in the skeletal muscles of mice following 7,8-DHF administration (Ahuja et al., 2022).

A potential cellular mechanism underlying the increased volume changes seen in the current study following 7,8-DHF administration could be spinogenesis which could in turn result in increased density of synapses. For example, 7,8-DHF was found to rescue synaptic loss in the CA1 of the 5XFAD AD mouse model (Zhang et al., 2014). The density of thin spines in the hippocampus of CaM/Tet-DT_A_ mice was also found to be increased (Castello et al., 2014). Dendritic loss was found to be improved in the hippocampus along with increased α-amino-3-hydroxy-5-methyl-4isoxazolepropionic acid receptor (AMPAR) GluA1 and GluA2 subunits in Tg2576 mouse model (Gao et al., 2016). Chronic administration of 7,8-DHF was found to improve MWM memory in aged rats. Phosphorylated extracellular signal-regulated kinases 1 and 2 (pERK1/2) increased in the hippocampus along with increased spine density in older rats (Zeng et al., 2012). Another possible explanation for increased volume in certain brain regions could be enhanced neurogenesis. In one study increased numbers of NeU positive cells were found in the hippocampus of perimenopausal mice administered 7,8-DHF (Amin et al., 2020). Neurogenesis was also found to be increased in the hippocampus of APP/PS1 AD mice (Hsiao et al., 2014). However, this finding could potentially have been a result of the cohousing rather than the drug itself. Neurogenesis was also found to be increased along with increased pERK1/2 in the hippocampus of juvenile Down syndrome mice (Stagni et al., 2017) but this finding was not replicated in adult Down syndrome mice (Giacomini et al., 2019). Glutamate loss was found to be protected in the hippocampus of 5XFAD mice, but no increase in neurogenesis was observed in the hippocampus (Aytan et al., 2018). Most of the studies outlined above focused histology in the hippocampus region and where undertaken, short-term MWM memory was assessed. It should be noted that in our study we did not find significant ROI volume increases in the hippocampus, in fact we saw a decrease in volume. That said, the brains in our study were collected for imaging immediately following long-term spatial memory testing 28 days following training and 7,8-DHF administration. Further research is needed to determine the mechanisms underlying the volume changes seen in cortical and cerebellar brain regions. Future research would also be beneficial to investigate the effect of different concentrations of 7,8-DHF. Other studies have found that 7,8-DHF protects against oxidative stress (Cho et al., 2019) and rescues astrocytic deficits in the hippocampus (Wang et al., 2022).

In conclusion, we found that 7,8-DHF when administered during MWM training enhances long-term spatial memory. Changes in brain volume in a wide range of brain areas was observed but were not found to correlate with spatial memory enhancement. Gaining an overview of the brain regions affected by 7,8-DHF administration is critical when assessing it for therapeutic use.

## Data availability statement

The raw data supporting the conclusions of this article will be made available by the authors, without undue reservation.

## Ethics statement

All animal experiments were approved under the UK Animals (Scientific Procedures) Act 1986.

## Author contributions

FR-M conceived study, designed study, acquired the data, analysed the data, wrote the original manuscript, and revised the manuscript. AL, CT, AM-B, KM, and JL contributed to the data analysis and revised the manuscript. MT contributed to the data acquisition. HJ-B contributed to interpretation of the data and revised the manuscript. All authors gave final approval of the version to be published and agree to be accountable for all aspects of the work. All authors contributed to the article and approved the submitted version.

## Footnotes

^1^ https://mouse-imaging-centre.github.io/RMINC/
